# Developmental and stress regulation on expression of a novel miRNA, Fan-miR73, and its target ABI5 in strawberry

**DOI:** 10.1038/srep28385

**Published:** 2016-06-21

**Authors:** Dongdong Li, Wangshu Mou, Zisheng Luo, Li Li, Jarukitt Limwachiranon, Linchun Mao, Tiejin Ying

**Affiliations:** 1 Zhejiang University, College of Biosystems Engineering and Food Science, Zhejiang Key Laboratory for Agro-Food Processing, Hangzhou, 310058, People’s Republic of China

## Abstract

Abscisic acid (ABA) is a critical plant hormone for fruit ripening and adaptive stress responses in strawberry. Previous high-throughput sequencing results indicated that ABA-insensitive (ABI)5, an important transcription factor in the ABA signaling pathway, was a target for a novel microRNA (miRNA), Fan-miR73. In the present study, exogenous ABA treatment was found to accelerate fruit ripening through differentially regulating the transcripts of ABA metabolism and signal transduction related genes, including *NCED1*, *PYR1*, *ABI1*, and *SnRK2.2*. Expression of Fan-miR73 was down-regulated in response to exogenous ABA treatment in a dosage-dependent manner, which resulted in an accumulation of *ABI5* transcripts in the ripening-accelerated fruits. In addition, both UV-B radiation and salinity stress reduced the transcript levels of Fan-miR73, whereas promoted *ABI5* expression. Furthermore, high negative correlations between the transcriptional abundance of Fan-miR73 and *ABI5* were observed during ripening and in response to stress stimuli. These results enriched the possible regulatory role of miRNA involved in the post-transcriptional modification of ABI5 during strawberry ripening, as well as responses to environmental stresses.

Abscisic acid (ABA) plays important roles in a vast array of plant developmental processes, such as seedling germination, fruit development and ripening, and responses to stress stimuli^1^. The identification of the nucleocytoplasmic receptors contributed to the comprehensive elucidation of the ABA core signaling pathway, ABA-PYR/PYL/RCARs-PP2Cs-SnRK2s, in which PYR/PYL/RCARs change their conformation in the presence of ABA before binding to and inhibiting PP2Cs (type 2C protein phosphatase), which release SnRK2s to be actively phosphorylated and subsequently phosphorylate the transcriptional regulators^2^. Worthy to note, abscisic acid insensitive (ABI)5, a typical subfamily belonging to the basic domain/leucine zipper (bZIP) transcription factors, was a pivotal regulator in the promotion of ABA-dependent gene expression^2^,^3^. The ABI5 family genes were predominantly expressed in seeds and appear to be responsible for seed dormancy and seedling growth arrest^4^.

The transcription, translation and stability of ABI5 are controlled by various regulating mechanisms. With respect to post-translational regulation, the best-studied modifications of ABI5 are protein phosphorylation/dephosphorylation, ubiquitination, and sumolylation^5^. ABI5 is known to be phosphorylated by SnRK2s to become active before binding to an ABA-responsive element, conserved in the promoters of many ABA-induced genes. Some other regulators were also found to be involved in the modification of ABI5. For example, the Ser/Thr protein phosphatase6 in Arabidopsis dephosphorylates and destabilizes ABI5, resulting in an antagonistic action with SnRK2 kinases^6^. Conversely, a protein phosphatase2A-associated protein, TAP46, can stabilize the phosphorylated ABI5 by physically binding to it to prevent the removal of the phosphate^7^. Accumulation of ABI5 has been shown to be under the regulation of microRNA (miRNA)^2^,^4^,^5^. For example, the mutation of *hyl1* (HYPONASTIC LEAVES 1, also known as DOUBLE-STRANDED RNA BINDING PROTEIN1, a key protein involved in miRNA biosynthesis) led to an ABA hypersensitive phenotype which might be partially ascribed to the over-accumulation of the ABI5 transcription factor^8^.

The perception and signaling of ABA were important for strawberry fruit development and ripening^9^,^10^. Previously, silencing of a crucial ABA biosynthetic gene encoding the 9-cis-epoxycarotenoid dioxygenase (NCED)1 was found to delay fruit color formation. This could be rescued by exogenous ABA in wild-type fruits, but not in fruits containing RNA interference against an ABA receptor (magnesium chelatase H subunit)^9^. On the other hand, ABA homeostasis is regulated by the key degradation genes *CYP707A* encoding the ABA 8′-hydroxylases which are critical in the predominant ABA catabolic pathway^11^. For instance, the ripening and fruit coloring were promoted in *PacCYP707A2*-RNAi-treated sweet cherry fruits^12^. With respect to ABA signaling, an ABA receptor PYR1 was proven to play a positive role in strawberry fruit ripening through regulation of a variety of ABA-responsive genes, including *ABI1* and *SnRK2*^13^. Moreover, it was found that gene silencing of an important PP2C, *ABI1*, could promote strawberry ripening^14^. Regarding the downstream of ABA signaling in strawberry ripening, the number of studies is still limited. Recently, the role of the R2R3-MYB10 transcription factor was characterized in strawberry receptacle ripening. Expression of this transcription factor was activated by ABA treatment, and transient silencing and transcriptomic analyses indicated that MYB10 might be a crucial regulator in ABA-induced anthocyanin biosynthesis^15^. Despite the well-elucidated functions of ABA signaling pathway in strawberry fruit ripening, the specific regulatory mechanisms driving expressions of these components remains elusive.

MiRNAs are a class of endogenous non-coding small RNAs of ~21 nucleotides in length. In the past few years, numerous miRNAs, including conserved and novel miRNAs, have been identified to play important roles in a vast array of plant developmental processes by next generation sequencing technologies^16^,^17^. Whereas the number of the miRNAs in the database boosted quickly, the specific functions of the majority of the predicted miRNAs are still largely unknown and remain to be further elucidated experimentally. As we known, the high complementarity of miRNAs to their target mRNAs commonly resulted in down-regulation of gene expression^18^. It has been proven that miRNAs could target ABA signaling related components to regulate their expression in adaptive stress responses. For instance, miR1432 was recently found to target an ABRE-binding factor BZ-1, and its down-regulated expression might be responsible for higher drought tolerance in sugarcane^19^. Up-regulated or down-regulated expression of miR1029 which targeted a DREB transcription factor were observed in wheat in response to osmotic or salinity stress, respecitvely^20^.

In addition to stress response, an increasing number of studies have recently revealed that miRNAs acted as important mediator in plant development through the post-transcriptional regulation on phytohormone metabolism and/or signaling. For example, miR160 was shown to repress the level of the transcription factor *ARF10* during seed germination and post-germination stages in *Arabidopsis thaliana*^21^. MiR167-directed repression of ARF8a and ARF8b was also found to be involved in soybean nodulation^22^. *IAA28*, a negative component in the auxin signaling pathway, is cleaved by miR847 to control lateral organ development in Arabidopsis^23^. Moreover, a novel miRNA, miR10515, also regulates IAA metabolism^24^. These studies indicated that miRNAs were effective regulators in hormonal signaling by targeting various components of those pathways. However, information pertaining to miRNA-directed regulation on ABA signaling is scarce. Previously, ABA was shown to induce miR159 and lead to transcript degradation of two MYB transcription factors during Arabidopsis seed germination^25^. In the present study, we report that Fan-miR73, a novel miRNA identified in a previous high-throughput sequencing experiment, targeted ABI5. The expression level of Fan-miR73 was modulated by exogenous ABA and subsequently altered ABI5 expression, which might be important for the acceleration of ABA-induced fruit ripening. The interrelation between Fan-miR73 and ABI5 was further investigated in response to salt stress and UV-B radiation.

## Results

### The identification of a novel miRNA and its target

**Figure 1:**
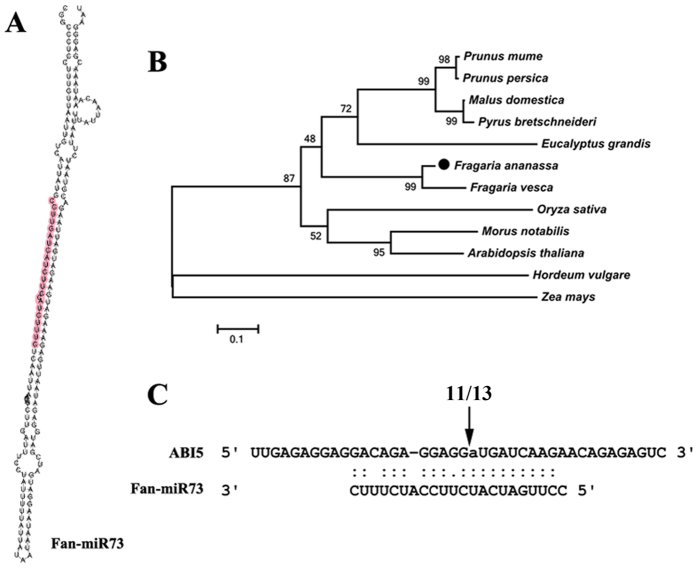
Fan-miR73 and its target ABI5. (**A**) The secondary structure of Fan-miR73, the purple sequence shows the mature miRNA on the 5′ arm. (**B**) Phylogenetic tree of the ABI5 transcription factor based on nucleotide sequence similarity. (**C**) Previously degradome result indicated miRNA-ABI5 duplex, which was validated by 5′-RACE analysis. Out of the13 detected fragments, 11 coincided with the predicted cleavage position between the 10^th^ and 11^th^ nucleotides.

More than 500 novel miRNAs in *Fragaria ananassa* were identified in our previous high-throughput sequencing results, among which Fan-miR73 was of special interest because its target mRNA, *ABI5*, encodes an important transcription factor in the ABA signaling pathway. The secondary structure of the pre-miRNA of Fan-miR73 is shown in Fig. 1A, and the colored sequence indicates the mature miRNA whose cluster appeared at the 5′ end. *FaABI5* was sequenced and blasted in NCBI, and a phylogenetic tree was generated by aligning with other similar *ABI5* genes using MEGA 5.10 program with a neighbor joining algorithm (Fig. 1B). The phylogenetic tree showed that *FaABI5* was highly related to the ABI5 gene from *Fragaria vesca*. Our previous degradome sequence has revealed the specific cleavage site of Fan-miR73 with its target mRNA ABI5 corresponding to the highly complementary sequences. This finding was further experimentally verified by 5′RACE which showed that out of the13 detected fragments, 11 were exactly found to have the same cleavage site between the 10th and 11th nucleotides of Fan-miR73 (Fig. 1C). These results identified the close interaction between Fan-miRNA and transcription factor *ABI5*. Thereafter, we want to further investigate the role of Fan-miR73 participated in certain ABA-involved processes, possibly by regulating the expression of ABI5.

### The expression profile of Fan-miR73 and ABA pathway genes during strawberry fruit ripening

**Figure 2:**
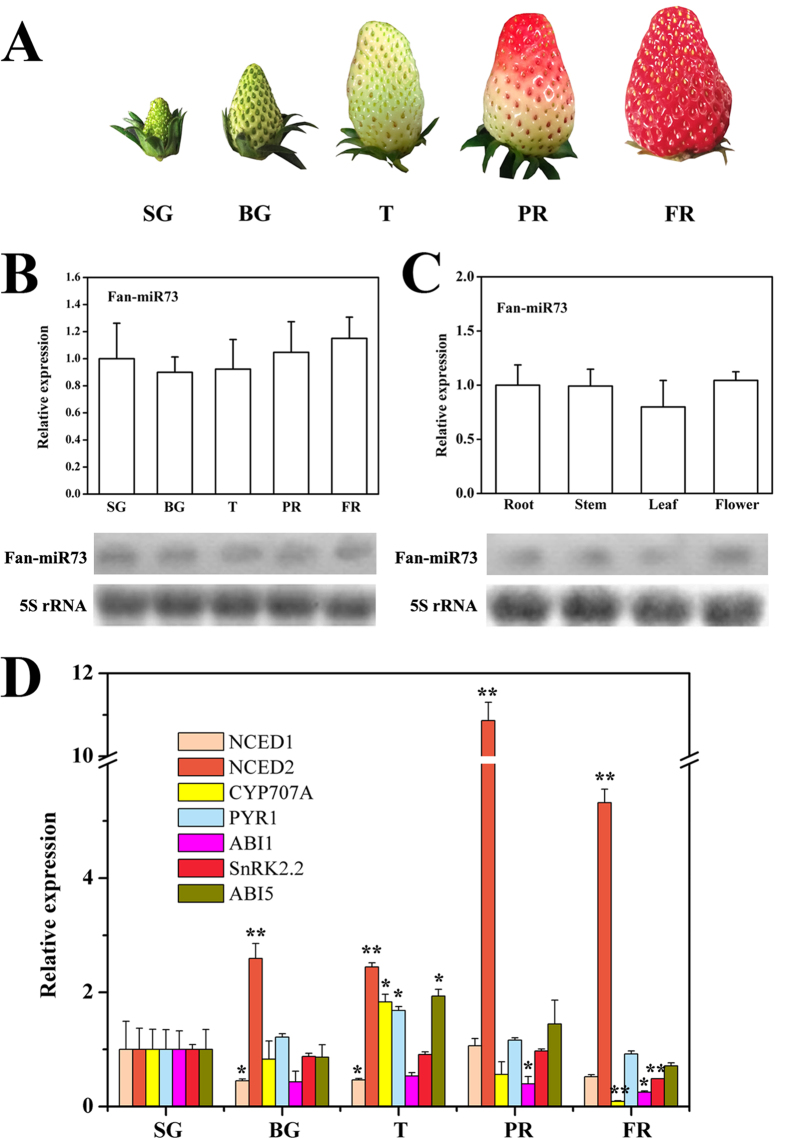
ABA signaling and expression profile of Fan-miR73 during strawberry fruit ripening. (**A**) The five ripening stages designated in the present study: SG, small green (7 DPA); BG, big green (14 DPA); T, turning (24 DPA); PR, partial ripening (28 DPA); FR, full ripening (30 DPA). (**B**) The expression profile of Fan-miR73 in fruits at different stages. (**C**) Fan-miR73 expression in different tissues and northern blotting analysis. 5S rRNA served as a loading control. (**D**) The expression profile of ABA metabolism and signaling related genes in fruits at different stages. DPA, days post-anthesis. In the bar-diagram the data expressed as mean ± SD of three replicates. *P < 0.05, **P < 0.01.

The developmental process of strawberry fruit were divided into 5 stages (Fig. 2A), that is, the small green (SG), big green (BG), turning (T), partial ripening (PR) and full ripening (FR) stages. Fan-miR73 was detected during the whole ripening process as revealed by northern blot analysis; meanwhile, the expression of Fan-miR73 was quantified by real-time PCR which showed a slight decrease at BG stage followed by a gradual increase afterwards as fruit ripening (Fig. 2B). The presence of Fan-miR73 was also verified in tissues of root, stem, leaf and flower (Fig. 2C). The expression levels of Fan-miR73 were relative consistent across tissues, though it was expressed less in the leaves.

ABA plays a vital role during strawberry fruit ripening, thus the expression of genes encoding enzymes in ABA metabolism and components in the ABA signaling pathway was determined to obtain a better understanding of the close interrelation between the novel miRNA and ABI5. As shown in Fig. 2D, *NCED1* expressed at a low level with a slight decrease during fruit ripening; while the expression of *NCED2* was significantly enhanced after SG stage and was almost 11-fold level higher at PR stage compared with that at SG stage. These results indicated that *NCED2* might be critical for the biosynthesis of ABA during ripening in strawberry. ABA homeostasis was tightly controlled by both biosynthesis and catabolism. In the present study, *CYP707A* was expressed highly at T stage and then decreased dramatically in later stages. In addition, the expression of the ABA receptor *PYR1* increased to approximately two fold at T stage, thereafter it decreased slowly until the FR stage. A similar trend of expression was observed for *ABI5*. In contrast, *ABI1*, one of the PP2Cs, was expressed at very low levels during all developmental and ripening stages. With regard to *SnRK2.2*, a remarkable lower level was only observed at the FR stage.

### Effects of exogenous ABA treatment on expression patterns of Fan-miR73 and ABI5 during ripening in strawberry fruit

**Figure 3:**
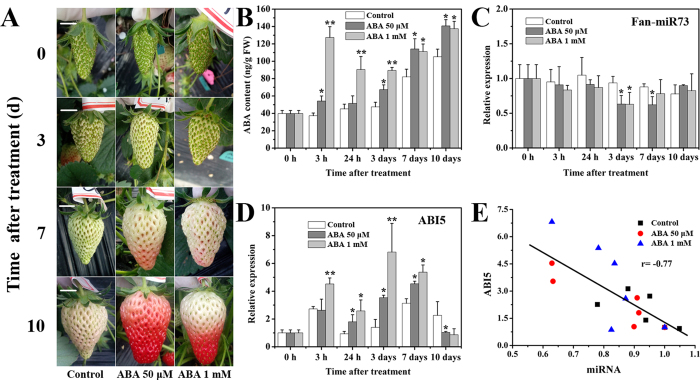
ABA accelerated strawberry fruit ripening. (**A**) Morphological changes of strawberry fruits in response to exogenous ABA treatment at different dosages (1 mM and 50 μM). On day 0, big green fruits (14 DPA) were dipped in ABA solutions at concentration of 1 mM and 50 μM. Control fruits were dipped in distilled water. Samples were taken immediately after dipping, and subsequently after 3 h, 24 h, 3 days, 7 days and 10 days. Bar = 1 cm. (**B**) The changes of ABA content in the fruits in response to different treatments. (**C**) The expression changes of Fan-miR73 in response to different treatments. (**D**) The expression changes of *ABI5* in response to different treatments. (**E**) The correlation rate between the expression of *ABI5* and Fan-miR73. Values are means ± SD of three independent experiments. *P < 0.05, **P < 0.01.

ABA acted as an important hormonal regulator during strawberry fruit development and ripening, which was confirmed by the morphological changes in response to different dosages of ABA treatment (Fig. 3A). The results showed that 1 mM ABA markedly accelerated the ripening process, whereas faster acceleration was observed in fruits treated with 50 μM ABA. Meanwhile, ABA content increased during fruit ripening in all treatments but to different extents (Fig. 3B). ABA treatment enhanced the accumulation, where an intensively high level of ABA content in fruits treated with 1 mM ABA was observed in the first 3 h after the treatment. This might be due to higher-concentration absorption in the first hours just after exogenous ABA treatment. Since the target mRNA of Fan-miR73 was involved in the ABA signaling pathway, it is interesting to investigate the specific response of this novel miRNA to ABA treatment. The expressions of Fan-miR73 decreased slightly during fruit ripening, however, ABA treatment lead to a much lower level especially on day 3 (Fig. 3C). This reduction of Fan-miR73 might be critical for the release for *ABI5* transcripts to regulate fruit ripening process. *ABI5* was expressed highly in ABA-treated fruits, in which the expression was also induced 3 h and 24 h later after the treatment (Fig. 3D). Nevertheless, the expression of *ABI5* in fruits on day 10 was reduced to the same level as that on day 0, indicating the importance of ABI5 in the regulation of fruit ripening process. MiRNAs were generally considered to down-regulate the transcript abundance of their targets. In the present study, a highly negative correlation rate (r = −0.77) between Fan-miR73 and *ABI5* expression was observed in fruits in response to ABA treatments (Fig. 3E). These results indicated that Fan-miR73 might participate in the ripening process of strawberry fruit by its reduction in expression so that *ABI5* was free to be expressed and to activate the transcripts of ABA responsive genes.

### Effects of exogenous ABA treatment on expression patterns of genes involved in ABA metabolism and signaling pathway

**Figure 4:**
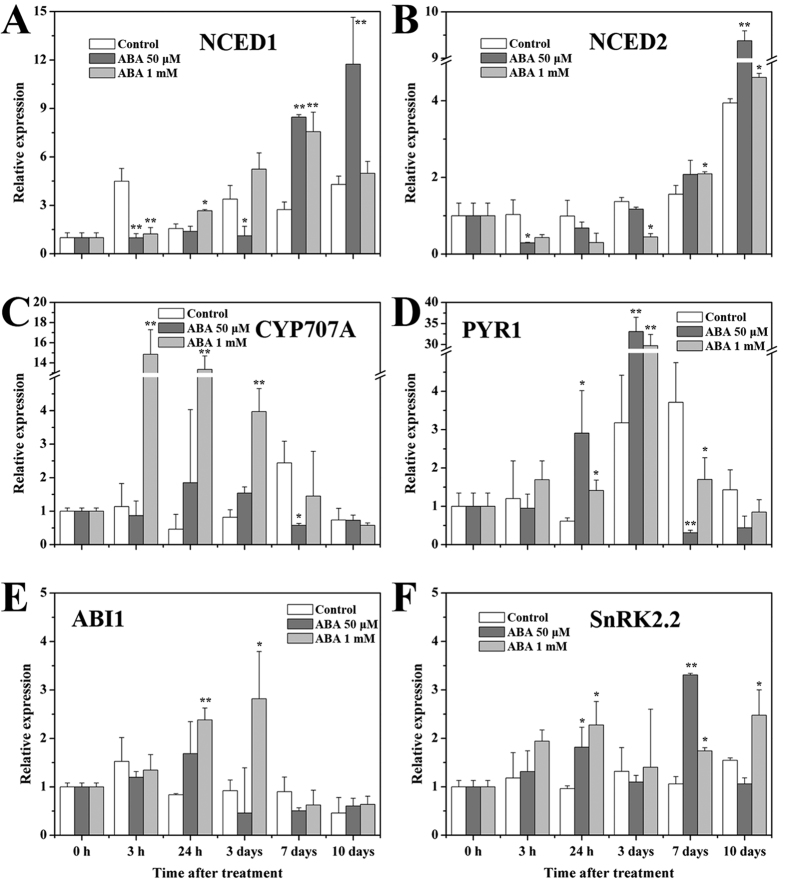
Effects of exogenous ABA treatment on the expression of ABA metabolism and signaling related genes. (**A**) NCED1. (**B**) NCED2. (**C**) CYP707A. (**D**) PYR1. (**E**) ABI1. (**F**) SnRK2.2. In the bar-diagram the data expressed as mean ± SD of triplicates. *P < 0.05, **P < 0.01.

For better illustration of the inter-relationship between the novel miRNA and the ABA signaling component ABI5, genes involved in ABA metabolism and signaling pathway were also investigated by qRT-PCR. The results showed that the expression of *NCED1* was significantly enhanced by ABA treatment especially at the late period of ripening (Fig. 4A). It was approximately 2-fold higher in fruits treated with 50 μM ABA than that in control fruits on day 7 and 10. Nevertheless, the expression levels of *NCED2* were down-regulated by ABA treatment in the first 3 days, but significantly higher level was observed in 50 μM ABA-treated fruits on day 10 (Fig. 4B). Expression of *CYP707A* decreased slightly during ripening before starting to increase at T stage (on day 7) in the control fruits (Fig. 4C). The expression of *CYP707A* was markedly up-regulated by 15 fold 3 h after the treatment in fruits treated with 1 mM ABA, and to a lesser extent in fruits treated with 50 μM ABA. These results indicated that exogenous ABA treatment enhanced the accumulation of ABA content in the fruits, which might mainly be related to the corresponding stimulated higher expressions of ABA biosynthetic genes. The regulation of ABA content balance by *CYP707A* might also be crucial for the development and ripening of fruit. In addition, *PYR1*, one of the most important receptors in ABA signaling pathway, was expressed highly as fruit ripened with a maximum level on day 7 before decreasing remarkably in control fruits (Fig. 4D). The expression profile of *PYR1* was accelerated by ABA treatment and the expression was more than 10-fold higher in ABA-treated fruits on day 3 followed with a dramatic decrease. On the contrary, expression of *ABI1* in control fruits decreased gradually during ripening, whereas it was up-regulated by 1 mM ABA treatment in the first 3 days then decreased to a very low level during the later period (Fig. 4E). Changes of expression of *SnRK2.2* were minor in control fruits during ripening, however, its expression was stimulated by ABA treatment in the first day and decreased slightly before another increase on day 7 (Fig. 4F). These results revealed that ABA signaling transduction might be important for the ABA-stimulated strawberry fruit ripening process.

### Expression profiles of Fan-miR73 and ABI5 and their correlation in response to stress stimuli

**Figure 5:**
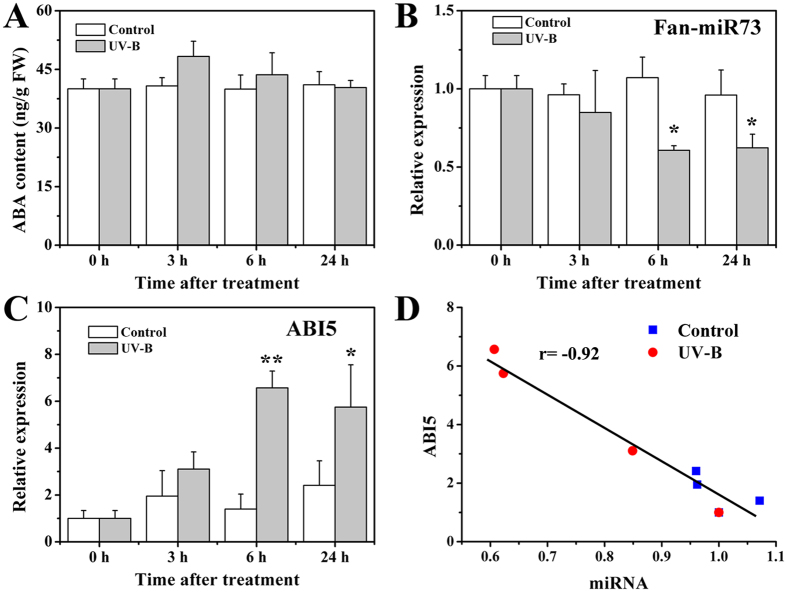
Effect of UV-B radiation on the levels of (**A**) ABA content, (**B**) Fan-miR73 expression, and (**C**) *ABI5* expression. (**D**) The correlation rate between the expression of *ABI5* and Fan-miR73. The data was expressed as mean ± SD of three replicates. *P < 0.05, **P < 0.01.

ABA plays an active role in stress responses. Stresses, including UV-B radiation and salt stress, were applied to evaluate changes in expression of Fan-miR73 and *ABI5*, and their correlation. Results showed that ABA content increased about 15% during 3 h after UV-B treatment, but no significant differences were observed at other time points (Fig. 5A). Nevertheless, remarkable decrease was observed in Fan-miR73 expression in response to UV-B radiation (Fig. 5B). The expression of Fan-miR73 was down-regulated more than 40% by UV-B irradiation 6 h or later after treatment; however, the expression of *ABI5* was up-regulated by UV-B radiation with a maximum level of approximate 6 fold at 6 h after treatment (Fig. 5C). The correlation rate between expression of Fan-miR73 and ABI5 was evaluated (Fig. 5D), and the result showed that there was a high negative regulating relationship (r = −0.92) between Fan-miR73 and ABI5.

**Figure 6:**
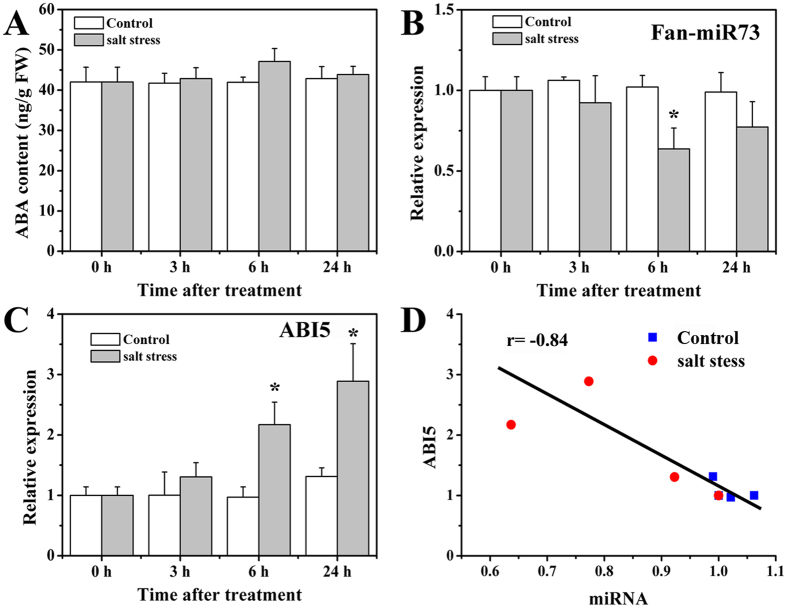
Effect of salt stress on the changes of (**A**) ABA content, (**B**) Fan-miR73 expression, and (**C**) *ABI5* expression. (**D**) The correlation rate between the expression of *ABI5* and Fan-miR73. The data was expressed as mean ± SD of triplicates. *P < 0.05, **P < 0.01.

Furthermore, we investigated the expressions of Fan-miR73 and ABI5 under salt stress. Changes of ABA content in the fruits were minor with a slight increase at 6 h after the treatment (Fig. 6A), whereas significantly down-regulated expression of Fan-miR73 was observed in fruits under salt stress at 6 h after the treatment (Fig. 6B). Conversely, the expression of *ABI5* was gradually up-regulated after salt stress and it reached about 3 fold at 24 h after treatment (Fig. 6C). The correlation rate (r = −0.84), slightly lower than that in UV-B treated fruits, also indicated that a highly negative relationship existed between expression of Fan-miR73 and *ABI5* (Fig. 6D). Taken together, these results suggest that Fan-miR73 might be actively regulated during stress responses as a decrease in its expression might facilitate the activation of ABA signaling.

## Discussion

The base-complementary targets of numerous miRNAs are commonly recognized to be transcription factors, among which, many are involved in hormonal signaling. More importantly, such interrelation between miRNAs and hormonal signaling components may be vital for fruit development. For instance, miR160 was identified to target and regulate auxin transcription factors during the development of tomato and pear fruit^26^,^27^. In the present study, a novel strawberry miRNA, registered as Fan-miR73, was found to target the key transcription factor ABI5 in the ABA signaling transduction pathway validated by 5′RACE in this study (Fig. 1). The results indicated that this miRNA might mediate ABA’s signaling by guiding cleavage of its target mRNA, thus regulating the transcript abundance of ABI5. Similarly, another key factor in ABA signaling pathway, *ABI4*, was found to be tightly regulated at the post-transcriptional level^28^. Thus it was possible that ABI5 might be under multilevel control after its transcription, and miRNA might participate at the post-transcriptional level to guide *ABI5* degradation.

The critical role of ABA in the regulation of strawberry fruit ripening including genes encoding enzymes related to its biosynthesis and catabolism and components involved in the central signal complex of PYR1/PYLs-PP2Cs-SnRK2s have been well studied^9^,^11^,^14^. Consistent with these studies, our results revealed that ABA metabolism and signal transduction related genes, including *NCED2*, *CYP707A*, *PYR1* and *ABI1* were important for the fruit ripening process (Figs 2D and 4). The results also raised the possibility that ABI5, as an important nuclear transcription factor at the down-stream of ABA signaling pathway, might be responsible for the expression activation of ABA-responsive genes for fruit development and ripening. Moreover, small RNAs deep sequencing has elucidated that fruit development and ripening was under the regulation of conserved and non-conserved miRNAs, such as in tomato^26^,^29^, pear^27^ and date palm^30^. On the other hand, miRNAs were observed to be regulated by ABA^31^. Therefore, it was likely that miRNA participated in the ABA-induced strawberry fruit ripening in the present study. Furthermore, the high negative correlation rates between ABI5 and Fan-miR73 indicated that ABI5 might be closely regulated by Fan-miR73 at the post-transcriptional level.

ABA plays an irreplaceable role in the adaptive stress responses, in which ABI5 is one of the diverse transcriptional activators in ABA signaling to enhance stress tolerance for plants^32^,^33^. Concurrently, increasing evidence has revealed that miRNAs played essential roles in biotic and abiotic stress adaptation by the regulation of miRNA expression correlated with the up- or down-regulated expression level of their target mRNAs^34^, among which many were found to be transcription factors^35^. For instance, the NAC, MYB, and MAPK families were of great importance in cotton to combat drought and salinity stress, and many conserved and novel miRNAs were identified to target these transcription members^35^. Sit-miR171b, which targets an auxin response factor, was significantly down-regulated under dehydration and salt stresses in a stress susceptible cultivar^36^. In contrast, sly-miR160 and miR167 were remarkably up-regulated in tomato fruits infected with *Cucumber mosaic virus*^37^. Our results indicated reduction in Fan-miR73 expression during the first 24 hours after stress treatment might contribute to the degradation-release of ABI5, which subsequently acted as an important activator to induce stress tolerance-related genes. Moreover, high negative correlation rates between the expression of ABI5 and Fan-miR73 also suggested that the regulatory mechanism might function in response to salt and UV-B stress (Figs 5 and 6). ABA is critical for the response of plants to high doses of UV-B radiation which is different from the strategy when encounter to low doses^38^. In the present study, enhanced UV-B radiation was applied to evaluate the regulatory relation between Fan-miR73 and ABI5. Although only a slight increase of ABA was observed in the UV-B-treated fruits in the first three hours, the expression of ABI5 was markedly up-regulated during the 24 hours, and simultaneously Fan-miR73 significantly down-regulated (Fig. 6). These results indicated that ABI5 is an important ABA-dependent transcription factor for stress response. Our study might help to enrich the regulatory network of hormones in response to stress, and elucidate the involvement of miRNAs in this process. Nevertheless, our results only revealed the possibility of Fan-miR73 being critical for ABA-dependent stress adaptation is high. Therefore, further genetic and molecular evidence including loss of function and overexpression analyses are needed in order to obtain a better understanding of the role of Fan-miR73 and its relationship with ABA.

In summary, a novel strawberry miRNA, Fan-miR73, was identified to target mRNA of the *ABI5* transcription factor which was an important component in ABA signaling pathway. Our results showed that Fan-miR73 was down-regulated by exogenous ABA treatment and might be responsible for the ABA-accelerated fruit ripening. In addition, the results indicated that the reduction of Fan-miR73 might be critical for fruits to cope with salt and UV-B stress, likely by releasing the post-transcriptional degradation effect on ABI5. Our study may help to enrich the possible regulatory role of miRNAs in ABA signaling at the post-transcriptional level during strawberry fruit ripening and stress responses.

## Materials and Methods

### Plant materials

Strawberry (*Fragaria* × *ananassa* Duch. ‘Toyonoka’) plants were planted in commercial greenhouses with 60–80% relative humidity and under cycles of 14 h light at 25 °C followed by 10 h dark at 20 °C. The developmental process of strawberry fruit has been divided into 5–7 stages in other species^14^,^39^,^40^, however, the white and initial red stages could not be easily distinguished in the ‘Toyonoka’ cultivar. Therefore, the two stages were combined and defined as turning stage. Thus, in this study, the strawberry fruits from five developmental stages were harvested, including small green (SG, approximate 7 days post-anthesis (DPA)), big green (BG, about 14 DPA), turning (T, 24 DPA), partial red (PR, 28 DPA), and full red (FR, 30 DPA), as described by Chen *et al*.^41^. The fruits were immediately frozen in liquid nitrogen after the calyx and peduncle were excised before storage at −80 °C. Roots, stems, leaves and flowers were collected from more than ten strawberry plants (about four-month old), rinsed with distilled water, frozen and stored for analysis.

### Northern blotting and cleavage site validation

We used different tissues including roots, stems, leaves and flowers for Northern blotting analyses. Small RNAs were firstly extracted from fruits at different developmental stages with a mirVana miRNA isolation kit (Ambion). The following procedures were conducted in accordance with Kong *et al*.^24^ with a slight modification where a 3′-DIG labeled LNA probe (Exiqon) was used for the hybridization. To examine cleavage site of predicted target mRNA ABI5, 5′ RACE (rapid amplification of cDNA ends) experiments were conducted with the SMARTer RACE cDNA Amplification Kit (Clontech, USA) according to the manufacturer’s protocol. PCR products were cloned into the pGEM T-Easy vector (Promega) and subsequently sequenced.

### RNA extraction and quantitative real-time PCR

The total RNA was isolated by using RNAiso Plus (TaKaRa, Japan) following the manufacturer’s protocol. The total RNA was extracted from fruits in response to ABA treatment, UV-B irradiation and salt stress, respectively. The RNA quantity was measured with a Nanodrop spectrophotometer (Nano Drop 2000, Thermo-Fisher scientific Inc., Wilmington, DE, USA). The RNA was reverse-transcribed using a RNA PCR kit (code DRR047A, TaKaRa) according to the manufacturer’s instructions, and the synthesized cDNA was used for qRT-PCR analysis which was performed on an ABI Step One RT-PCR system. The relative mRNA expression levels were quantified using SYBR^®^ Premix Ex Taq^TM^ (TaKaRa, Dalian, China) with *FaACTIN* as a reference gene. The results were calculated with the 2^−ΔΔCT^ method. Three biological and three technical replicates were conducted for each sample. Primers were listed in Supplemental Table S1.

### Quantitative RT-PCR analysis of Fan-miR73

The total RNA was extracted separately from each tissue and different fruit stages for miRNA quantitative RT-PCR analysis. A MicroVana miRNA isolation kit (Ambion) was used following the manufacturer’s protocol. The cDNA was synthesized with miRNA-specific stem-loop primers which were provided by a local commercial company (Biosci, Hangzhou). The total volume was 20 μL for reverse-transcription, and the reaction was incubated for 30 min at 42 °C, and followed by 10 min at 70 °C. A 20 μL PCR reaction containing SYBR green mix was incubated at 94 °C for 1 min, followed by 40 cycles of 95 °C for 10 s, 58 °C for 10 s and 72 °C for 10 s. The detection was performed on an Agilent Stratagene Mx3005P system. For each PCR product, the melting curve was analyzed to avoid non-specific amplification. The relative quantity was calculated using the 2^−ΔΔCT^ method with 5.8S as an internal control. All the primers used are listed in Supplemental Table S1. Three biological and three technical replicates were conducted for each sample.

### ABA treatment

For ABA solution preparation, ABA was firstly dissolved in a small volume of ethanol and then diluted with deionized water to concentrations of 50 μM and 1 mM (2% ethanol v/v total volume). Fruits at BG stage were dipped on the vine in ABA solutions for 1 min at 25 °C. Control fruits were dipped in 2% ethanol. Samples of control were taken immediately after dipping, and subsequently all samples of different treatments were taken after 3 h, 24 h, 3 days, 7 days and 10 days. Three replicates were conducted.

### UV-B and salt stress treatment

Fruits at BG stage were irradiated on vine with UV-B light, using two UV-B lamp tubes (TL 20W/01 RS with a spectral peak at 311 nm, Philips). The lamps were installed closely parallel to each other, and equipped with a plastic cover to protect the operator. The irradiation intensity was measured by a portable digital radiometer (TAINA TN-2340, TAINA Co., Ltd., Taiwan, China) and fixed at 10 ± 0.3 W/m^2^ by adjusting the vertical distance between the lamps and the fruits. The UV-B lamps were allowed to stabilize for at least 30 min prior to treatment. The irradiation dose applied in this study was 10 kJ/m^2^ through controlling the UV-B light exposure time. At the middle of the irradiation time, strawberry fruits were rotated gently by 180° horizon to ensure uniform irradiation. The daylight was not eliminated during UV-B radiation process. Fruits only exposed to daylight were set as controls. Samples were taken immediately, and after 3 h, 6 h and 24 h. Experiment of salt stress was carried out according to reports by Christou *et al*.^42^ and Garriga *et al*.^43^. Briefly, 12 plants (about four-month-old) with similar size were selected and cultivated individually in plastic pots, which were filled with sand and Hoagland nutrients supplying solution. The plants were allowed to acclimate for 20 days before the treatment, in which the nutrients solution was refreshed every 5 days. Finally, on the fourth nutrient refreshment, 6 plants were extra-supplied with 100 mM NaCl. Fruits were sampled immediately (0 h), and after 3 h, 6 h and 24 h of salinity stress.

### ABA content determination

Approximately 1 g of fruit was ground in 8 mL 80% methanol (v/v) for ABA extraction. Extracts were centrifuged at 9,000 *g* for 15 min, filtered and concentrated to less than 1 mL under vacuum. For polar compounds elution, the concentrated liquid was loaded to a Sep-Pak C_18_ cartridge (6 mL/500 mg, Waters Corporation, USA) which had been pre-treated with 15 mL 100% methanol and 15 mL 0.4% acetic acid. ABA was eluted from the Sep-Pak by 15 mL 10% (v/v) methanol −0.4% acetic acid. The eluates were dried under vacuum at 35 °C and the residues were dissolved in 1.5 mL 50% (v/v) methanol −0.4% acetic acid solution. After filtered through 0.22 μm membrane, ABA content measurement was performed on an Agilent 6460 Triple quadruple LC/MS system (Agilent Technologies Inc., USA) with a ZORBAX Eclipse XDB-C18 column (2.1 × 150 mm, 3.5 μm, Agilent Technologies Inc., USA) as previously described by Chen *et al*.^44^.

### Statistical analysis

Results were expressed as mean ± standard deviation (SD). All statistical analyses were performed with SPSS (SPSS Inc., Chicago, IL, USA). Data were analyzed using ANOVA, and the means were compared by the least significant difference (LSD) at significance levels of *P < 0.05 and **P < 0.01.

## Additional Information

**How to cite this article**: Li, D. *et al*. Developmental and stress regulation on expression of a novel miRNA, Fan-miR73, and its target ABI5 in strawberry. *Sci. Rep.* **6**, 28385; doi: 10.1038/srep28385 (2016).

## Supplementary Material

Supplementary Information
