# Supplementary material for: Developmental and stress regulation on expression of a novel miRNA, Fan-miR73, and its target ABI5 in strawberry
Source: Sci Rep. 2016 Jun 21;6:28385. doi: 10.1038/srep28385 (PMC4914977; doi:10.1038/srep28385)
Supplement: Supplementary Information [file srep28385-s1.pdf]

Supplementary information for

Developmental and stress regulation on expression of a novel miRNA, Fan-miR73, and its target ABI5 in strawberry

Dongdong Li, Wangshu Mou, Zisheng Luo, Li Li, Jarukitt Limwachiranon, Linchun Mao, Tiejin Ying

Table S1: Primers used in this study.

Primer	Sequence 5'-3'
Fan-miR37-RT	GTCGTATCCAGTGCAGGGTCCGAGGTATTCCGACTGGATACGACgaaaga
Fan-miR37-F	CCAGCTTGATCATCTTCC
Fan-miR37-R	AGCAGGGTCCGAGGTATTC
5.8S-RT	GTCGTATCCAGTGCAGGGTCCGAGGTATTCCGACTGGATACGACgtgtga
5.8s-F	CACCTGTTACCATCCCGA
5.8s-R	AGCAGGGTCCGAGGTATTC
Northern blot probe	GAAAGATGGAAGATGATCAAGG/3Dig_N/
RACE Oligo-adaptor	CGACTGGAGCACGAGGACACTGACATGGACTGAAGGAGTAGAAA
RACE-FP1	GGTGTAGGATTCGGGTTTCAGCA
RACE-FP2	GAAGTTGCAAAGCTAAAAGAGGAGAA
RACE-RP1	CCTGCTCGCCGATCCCCATA
RACE-RP2	CTACAGCACAAGACACATGATCCTTA
NCED1-FP	ACGACTTCGCCATTACCG
NCED1-RP	AGCATCGCTCGCATTCT
NCED2-FP	TACGGGGACAGGAAGTTCGGTG
NCED2-RP	TCAAACTCACGGCGTTCACAATCT
CYP707A1-FP	CTGTGACCTCGGAGTCGTGGG
CYP707A1-RP	TCCTCAGTTCTTCTTTGTAGCGGGT
PYR1-FP	AAACCTTGTTCCGCTCCT
PYR1-RP	CAATGGCAATCTCCCTCC
ABI1-FP	GTCGTGGCAAACAACCTG
ABI1-RP	TTCCACTGTATGACCTTCCCT
SnRK2.2-FP	GCACTTCCGTCCAAGAGTG
SnRK2.2-RP	AGGATATGTAGTGCTGGTAGATT
ABI5-FP	GGAGCTGGCAATGGTCG
ABI5-RP	AGGCCCGCCTTTCCTT
Actin-FP	TGGGTTTGCTGGAGATGAT
Actin-RP	CAGTAGGAGAACTGGGTGC
